# Correction: From lab to ocean: bridging swimming energetics and wild movements to understand red drum (*Sciaenops ocellatus*) behavior in a tidal estuary

**DOI:** 10.1242/jeb.253045

**Published:** 2026-07-13

**Authors:** Brendan J. Gibbs, James A. Strother, Clark R. Morgan, Daniele Pinton, Alberto Canestrelli, James C. Liao

There was an error in *J. Exp. Biol.* (2026) **229**, jeb251420 (doi:10.1242/jeb.251420).

The first name of author Daniele Pinton was spelt incorrectly (Danielle Pinton). The correct name appears above and both the online full-text and PDF versions of the article have been updated.

We apologise to the authors and readers for this error and any inconvenience it may have caused.

